# Vascular Ageing Features Caused by Selective DNA Damage in Smooth Muscle Cell

**DOI:** 10.1155/2021/2308317

**Published:** 2021-08-31

**Authors:** Ehsan Ataei Ataabadi, Keivan Golshiri, Janette van der Linden, Martine de Boer, Dirk J. Duncker, Annika Jüttner, René de Vries, Richard Van Veghel, Ingrid van der Pluijm, Sophie Dutheil, Suman Chalgeri, Lei Zhang, Amy Lin, Robert E. Davis, Gretchen L. Snyder, A. H. Jan Danser, Anton J. M. Roks

**Affiliations:** ^1^Division of Pharmacology and Vascular Medicine, Department of Internal Medicine, Erasmus MC, Rotterdam, Netherlands; ^2^Division of Experimental Cardiology, Department of Cardiology, Thorax Center, Erasmus MC, Rotterdam, Netherlands; ^3^Department of Molecular Genetics, Erasmus MC, Rotterdam, Netherlands; ^4^Department of Vascular Surgery, Erasmus MC, Rotterdam, Netherlands; ^5^Intra-Cellular Therapies Inc, 430 R 29th Street, Suite 900, New York, NY 10016, USA

## Abstract

Persistently unrepaired DNA damage has been identified as a causative factor for vascular ageing. We have previously shown that a defect in the function or expression of the DNA repair endonuclease ERCC1 (excision repair cross complement 1) in mice leads to accelerated, nonatherosclerotic ageing of the vascular system from as early as 8 weeks after birth. Removal of ERCC1 from endothelial alone partly explains this ageing, as shown in endothelial-specific *Ercc1* knockout mice. In this study, we determined vascular ageing due to DNA damage in vascular smooth muscle cells, as achieved by smooth muscle-selective genetic removal of ERCC1 DNA repair in mice (SMC-KO: SM22*α*Cre+ *Ercc1*fl/-). Vascular ageing features in SMC-KO and their wild-type littermates (WT: SM22*α*Cre+ *Ercc1*fl/+) were examined at the age of 14 weeks and 25 weeks. Both SMC-KO and WT mice were normotensive. Compared to WT, SMC-KO showed a reduced heart rate, fractional shortening, and cardiac output. SMC-KO showed progressive features of nonatherosclerotic vascular ageing as they aged from 14 to 25 weeks. Decreased subcutaneous microvascular dilatation and increased carotid artery stiffness were observed. Vasodilator responses measured in aortic rings in organ baths showed decreased endothelium-dependent and endothelium-independent responses, mostly due to decreased NO-cGMP signaling. NADPH oxidase 2 and phosphodiesterase 1 inhibition improved dilations. SMC-KO mice showed elevated levels of various cytokines that indicate a balance shift in pro- and anti-inflammatory pathways. In conclusion, SMC-KO mice showed a progressive vascular ageing phenotype in resistant and conduit arteries that is associated with cardiac remodeling and contractile dysfunction. The changes induced by DNA damage might be limited to VSMC but eventually affect EC-mediated responses. The fact that NADPH oxidase 2 as wells as phosphodiesterase 1 inhibition restores vasodilation suggests that both decreased NO bioavailability and cGMP degradation play a role in local vascular smooth muscle cell ageing induced by DNA damage.

## 1. Introduction

Cardiovascular disease (CVD) remains a leading cause of morbidity and mortality worldwide and has an enormous economic burden on healthcare systems [1]. Even with developments controlling the classical risk factors, such as smoking, high cholesterol, diabetes, or hypertension, cardiovascular problems remain a chief health issue. Ageing remains the largest risk factor for cardiovascular disease [2]. Ageing is defined as a time-dependent functional deterioration that affects most living organisms and causes advanced loss of physiological integrity, impaired organ function, and larger risk of premature death [3]. There are different factors that are associated with ageing or accelerate ageing of which accumulating DNA damage is a major one [1, 4, 5]. DNA damage can be induced by different sources like endogenous (e.g., generation of reactive oxygen species (ROS) and other oxidative reactions) or exogenous (e.g., UV and ionizing radiations) reactive agents that may cause hundred thousands of DNA lesions per cell per day [3, 6]. Because of the intricate network of DNA repair systems in our body, most of those lesions will be eliminated. However, some lesions are not repaired, and these persistent lesions can induce transcription problems, metabolic and signaling changes, and cellular senescence that cumulate with age. Depending on the mediator, extent of exposure, target cell, and individual differences in repair capacity, ageing shows different interindividual and organ-specific (segmental) rates of development, as observed in daily human life and animal models of ageing [7, 8].

Several genetically modified mouse strains have been generated that model accelerated ageing. These are based on a specific deficiency in DNA repair. Different DNA repair systems and components can be targeted to accelerate ageing, and among them, excision repair cross-complementation group 1 (ERCC1) is a protein that, when defective, affects several main DNA repair systems [9]. ERCC1-xeroderma pigmentosum (XP) F is a structure-specific protein complex which acts as an endonuclease enzyme involved in the repair of several types of DNA lesions, mainly bulky, helix-distorting lesions that are repaired by the nucleotide excision repair pathway, but also double-strand breaks and interstrand cross-links [10–12]. *Ercc1*-deficient mice have been used repeatedly by diverse groups to study human-like ageing features [13–16]. The *Ercc1^∆/-^* mouse is a convenient model to study vascular ageing and potential therapies. The ∆ allele is a truncation of the *Ercc1* gene by 7 amino acids of its C-terminus that disrupts the interaction between ERCC1 and XPF proteins and subsequently causes accumulation of DNA lesions in a progressive manner [7].

*Ercc1^∆/-^* mice have a short life span of around 24-28 weeks and display many human-like ageing features like neurodegeneration, osteoporosis, and liver, kidney, heart, and muscle dysfunctions that mostly start from the age of about 12 weeks [15, 17]. With regard to cardiovascular ageing, *Ercc1^∆/-^* mice show increased vascular stiffness and vascular wall thickness, increased blood pressure, and diminished macro- and microvascular relaxation that is mainly explained by reduced NO-cGMP pathway signaling, a major player in dysfunction of the aged cardiovascular system [17]. *Ercc1^∆/-^* mice display segmental progeria [18], which suggests that affected organs could be influenced by a local DNA damage process rather than a systemic one. Indeed, we have demonstrated that *Ercc1* knockout specifically in vascular endothelial cells (EC-KO) selectively affects endothelium-derived nitric oxide (NO) and leads to decreased end-organ perfusion, vascular leakage, and increased wall thickness [7]. However, vascular smooth muscle cells (VSMC) in *Ercc1^∆/-^* mice displayed an additional hyporesponsiveness to NO as compared to EC-KO mice [17]. Moreover, there is a rapid development of carotid artery stiffness in *Ercc1^∆/-^* mice which is absent in EC-KO [17]. These differences suggest that some of the ageing features in the *Ercc1^∆/-^* mice can be induced by DNA damage in VSMC [7, 17]. To address the question if a local VSMC DNA repair defect is critical for the specific changes in vascular function as observed in *Ercc1^∆/-^* mice, we investigated cardiovascular function in a mouse model with specific loss of *Ercc1* function in smooth muscle cells (SMCs). We focused on changes of NO-cGMP responsiveness since this is a major hallmark of ageing and DNA damage-related vascular dysfunction.

## 2. Methods

### 2.1. Animals

We evaluated the effect of SMC genomic instability on cardiovascular function in a mouse model with SMC-targeted deletion of *Ercc1*, based on crossbreeding of mice with floxed *Ercc1* gene with the B6.129S6-*Tagln^tm2(cre)Yec^*/J strain (SM22*α*creKI, Jackson Labs USA, stock no. 006878) [19]. SM22*α*creKI harbours a Cre recombinase-coding sequence under control of the endogenous transgelin, or smooth muscle 22 *α*-actin, promoter, which is the best-studied smooth muscle promoter. Expression of Cre recombinase in embryonal SMC and cardiac myocytes, a drawback of other SM22*α*Cre strains, is absent in this model [19, 20]. Apart from expression in vascular smooth muscle cells, there are reports suggesting promoter activation in platelets, adipocytes, and myeloid cells, and fully specific promoters for smooth muscle have hitherto not been identified [20]. To target the smooth muscle cells, various Cre recombinase models can be used. SM22Cre models have been used in this study. SM22*α* is an actin-binding regulatory protein involved in SMC contraction and serves as a marker to study a SMC-specific expression model [20].

As the *Ercc1*flox strain, we have used the FVB/N background-based transgenic mice generated by Melton et al. (Edinburgh, United Kingdom) [21]. SM22*α*Cre+/- female mice were crossed with *Ercc1*+/- male mice to generate SM22*α*Cre+/-::*Ercc1*+/- mice in a pure C57BL/6J background. This offspring was then crossed with *Ercc1*fl/fl mice in a pure FVB/N background to produce SM22*α*Cre+ *Ercc1*fl/- mice knockout mice (SMC-KO) in a C57BL6/FVB F1 hybrid background [22]. Thus, the *Ercc1* gene is fully inactivated for both alleles. Littermates (genotypes: SM22*α*Cre+ *Ercc1*fl/+) were used as controls. We also selected SM22*α*Cre- mice to exclude potential effects of Cre on vascular function.

Male and female mice were housed in individually ventilated cages, in a 12 h light/dark cycle, and fed normal chow and water *ad libitum*. SMC-KO developed weight difference compared with WT controls that at 6 months reached the threshold for termination of experiments on ethical requirements of the EU directive. Consequently, we decided to measure cardiac and vascular phenotyping features at 12-13 (early time-point: ET) and 22-23 weeks of age (late time-point: LT), when no visible clinical signs (except for the body weight loss) were evident. At the end of the experiment, mice were euthanized under deep anesthesia by exsanguination from the portal vein. We sacrificed the mice at the age of 14 and 25 weeks. Mice that were sacrificed at the age of 14 and 25 weeks old are referred to as earlier time-point (ET) and later time-point (LT), respectively, throughout this article. In total, for ET, 14 SMC-KO and 14 WT and for LT, 16 SMC-KO and 21 WT were included into the study. For each group, both genders were included with quite equal number and all the methods were performed with both genders. All animal procedures were performed at the Erasmus MC facility for animal experiments following the guidelines from Directive 2010/63/EU of the European Parliament on the protection of animals used for scientific purposes. All animal studies were approved by the National Animal Care Committee and the local administration within Erasmus University Medical Center Rotterdam.

### 2.2. Blood Pressure Measurement

Blood pressure (BP) was measured with a noninvasive method in conscious mice at both time-points using the tail cuff technique (CODA High-Throughput device, Kent Scientific). BP was measured on 5 consecutive days, and each session consisted of 30 measurement cycles per mouse. Acclimatization sessions were performed on the first 4 days, and the 5^th^ day was used as the main measurement for each mouse. BP values are reported as the average of all valid cycles recorded at day 5 [17].

### 2.3. Microvascular Vasodilator Function *In Vivo*

We assessed *in vivo* vasodilator function using Laser Doppler perfusion imaging (LDPI, Perimed, PeriScan PIM 3 System). Reactive hyperemia, defined as the hindleg perfusion that increases after temporary occlusion of the blood flow, was calculated. One day prior to LDPI in the left hindleg, hair was removed by hair-removal cream. The hindleg was kept still with the aid of a fixation device. After recording baseline perfusion for 5 minutes, blood flow was occluded for 2 minutes with a tourniquet. After releasing the tourniquet, blood flow was monitored for 10 minutes to observe its return to the postocclusion baseline and to record hyperemia. During all measurements, mice were under 2.8% isoflurane anesthesia, and body temperature was kept at 36.4-37.0°C by means of a heating pad with rectal temperature probe feedback. For each mouse, the maximum response to occlusion and the area under the curve (AUC) relative to the postocclusion baseline were calculated. Only the area above the baseline was considered, and values below the baseline were set to 0.

### 2.4. Mechanical Properties and Dimensions of the Vascular Wall

After removing the surrounding tissues, carotid arteries explanted from ET and LT mice were mounted in a pressure myograph (Danish Myograph Technology (DMT), Aarhus, Denmark) in calcium-free buffer (in mmol/L: NaCl 120, KCl 5.9, EGTA 2, MgCl2 3.6, NaH2PO4 1.2, glucose 11.4, and NaHCO3 26.3; pH 7.4), thus excluding measurements of both strain-induced contraction and an acute effect of PDE1 inhibition (PDE1 is relatively inactive in the absence of Ca^2+^). The intraluminal pressure of the carotid artery was increased stepwise by 10 mmHg starting at 0 mmHg and reaching 120 mmHg. Following each step, the vessels were allowed to equilibrate and then lumen and vessel diameters were measured and used to calculate strain and stress [23].

### 2.5. Cardiac Function

Mice were sedated with 4% isoflurane, intubated, and connected to a pressure-controlled ventilator. Anesthesia was maintained with 2.5% isoflurane, and body temperature was kept at 37°C [24]. Cardiac geometry and function were evaluated by performing 2-D-guided short-axis M-mode transthoracic echocardiography (Vevo 770 High-Resolution Imaging System, VisualSonics) equipped with a 35 MHz probe. Left ventricular (LV) external and internal diameters were traced, and subsequently, heart rate, fractional shortening, cardiac output, LV mass, and LV wall thickness were calculated using the Visual Sonics Cardiac Measurements Package. Cardiac volume was calculated from the measured diameters assuming a spheric shape. Echocardiography was performed at 13 and 24 weeks of age in mice that were sacrificed at LT, allowing us to obtain echocardiographic parameters for the same animal at two different time-points (corresponding to ET and LT). After completing echocardiography, mice were sacrificed and hearts were quickly excised and rinsed in ice-cold saline. Subsequently, LV was dissected from the right ventricle and atria and LV was weighed and stored for further analyses.

### 2.6. Histology in the Heart

Paraffin-embedded LV samples were cut into 4 *μ*m sections, deparaffinized, and stained to determine free wall myocardial collagen content and cardiomyocyte size. Collagen content was measured using picrosirius red staining according to the standard protocol. Images were analyzed using a quantitative image analysis system (BioPix iQ software, BioPix AB). Cardiomyocyte size was quantified by performing Gomori staining using the standard protocol. Images were analyzed using a quantitative image analysis software (Leica Qwin Plus V3).

### 2.7. *Ex Vivo* Vascular Function Assessment

Immediately after sacrificing the mice, the thoracic aorta and iliac arteries were carefully isolated from mice and kept in cold Krebs-Henseleit buffer (in mmol/L: NaCl 118, KCl 4.7, CaCl_2_ 2.5, MgSO_4_ 1.2, KH_2_PO_4_ 1.2, NaHCO_3_ 25, and glucose 8.3 in distilled water; pH 7.4). After removing the surrounding tissues, vessel segments of 1.5-2 mm length were mounted in small wire myograph organ baths (Danish Myograph Technology, Aarhus, Denmark) containing 6 mL of Krebs-Henseleit buffer oxygenated with 95% O_2_ and 5% CO_2_ at 37°C. The tension was normalized by stretching the vessels in steps until 90% of the estimated diameter at which the effective transmural pressure of 100 mmHg is reached [25]. Thereafter, the viability of the vessels was checked by inducing contractions with 30 and 100 mmol/L KCl. After reaching the maximum contraction induced by KCl, vessels were washed 4 times with a 5-minute interval. To evaluate vasodilatory responses, aortic and iliac segments were preconstricted with either 30 nmol/L of U46619 (a thromboxane A2 analogue) or 30 mmol/L KCl, resulting in a preconstriction corresponding with 50-100% of the response to 100 mmol/L KCl.

After reaching a contraction plateau on U46619, concentration-response curves (CRCs) were constructed with the endothelium-dependent vasodilator acetylcholine (ACh) at cumulative doses (10^−9^-10^−5^ mol/L). When the CRC of ACh was completed, the endothelium-independent vasodilator sodium nitroprusside (SNP, 0.1 mmol/L) was added. To evaluate the involvement of the NO-cGMP pathway, one segment was preincubated with NG-nitro-L-arginine methyl ester salt (L-NAME, 10^−4^ mol/L), an endothelial nitric oxide synthase inhibitor. To investigate the role of endothelium-dependent hyperpolarization (EDH), the small conductance Ca^2+^-activated K^+^ (SKCa) channel inhibitor apamin (100 nmol/L) and the intermediate conductance Ca^2+^-activated K^+^ (IKCa) channel inhibitor TRAM34 (10 *μ*mol/L) were added on top of L-NAME. We also evaluated the role of NADPH oxidase- (Nox-) dependent reactive oxygen species generation, in parallel rings, preincubated either with no inhibitor, apocynin (a broad spectrum Nox inhibitor, 10^−4^ mol/L), or GSK279503 (a selective Nox2 inhibitor, 6 *μ*mol/L). Inhibitors were given 10 minutes before U46619 except for apocynin (30 minutes) and GSK279503 (60 minutes).

In parallel rings, CRCs to NO-donor SNP (10^−11^-10^−4^ mol/L) were constructed. Rings were preconstricted with 30 mmol/L KCl to avoid bias of NO-cGMP responses by EDH. To avoid bias by intrinsic release of NO, segments were preincubated with L-NAME 10^−4^ mol/L added 20 minutes before preconstriction. To explore the contribution of PDE1 and PDE5, the most abundant cGMP-degrading PDEs in VSMC [1], segments were preincubated either with sildenafil 100 nmol/L (a selective PDE5 inhibitor) [16] or lenrispodun 100 nmol/L (a selective PDE1 inhibitor) [26] on top of L-NAME. Inhibitors were given 10 minutes before inducing preconstriction.

### 2.8. Molecular Analysis: Analysis of Plasma Cytokine Levels

For mouse plasma, protein levels of IL-1*β*, IL-2, IL-4, IL-6, IL-10, TNF-*α*, and IFN-*γ* were measured using a V-Plex Meso Scale Discovery (MSD) multiplex spot assay mouse neuroinflammation 1 panel. All samples were diluted at a ratio of 1 : 4 with diluent 41—provided in the MSD kit. Samples and standards were run in duplicate or triplicate according to manufacturer's instructions and analyzed with MSD Discovery Workbench software (Meso Scale Discovery, Gaithersburg, MD) at ITCI.

### 2.9. Quantitative Real-Time PCR

Total RNA was isolated from the abdominal aorta, heart, and kidney of LT mice using the RNeasy Mini Kit (Qiagen). The cDNA was synthesized from the total RNA using SuperScript IV First-Strand Synthesis System (ThermoFisher Scientific) according to the manufacturer's protocol. The cDNA was then amplified by quantitative real-time PCR on a QuantStudio 7 Flex Real-time PCR system (Applied Biosystems). Each reaction was performed in duplicate with TaqMan Universal Master Mix II (Applied Biosystems). The TaqMan Assay IDs and context probe sequences used for *Pde1a*, *Il-6*, and *Gapdh* are mentioned in Table S1. PCR cycling conditions were 50°C for 2 min, 95°C for 10 min, followed by 40 cycles of 95°C for 15 s and 60°C for 1 min.

To measure mRNA expression of *Ercc1*, *p16*, and *p21* in the abdominal aorta, each reaction was performed in duplicate with SYBR Green PCR Master Mix (UK, Applied Biosystems). PCR cycling conditions were 50°C for 2 min, 95°C for 2 min, followed by 40 cycles of 95°C for 15 s and 60°C for 1 min. *β*-Actin and *Hprt1* were used as household genes. Results from unreliable duplicates or melting curves were discarded. The relative amount of genomic DNA in DNA samples was determined as follows: relative quantification = 2(−ΔΔCt). The sense and antisense mouse primer sequences are mentioned in Table S2.

### 2.10. Statistical Methods

Data are presented as mean and standard error of the mean, unless otherwise indicated. Statistical analysis between the groups of single values was performed by unpaired, two-tailed *t*-test. Differences among the groups, depending on the number of variables, were analyzed by either one-way or two-way or three-way ANOVA followed by Bonferroni's post hoc test. Differences between CRCs were tested by general linear model for repeated measures (sphericity assumed). *p* values below 0.05 were considered as significant. Initial analysis was performed separately in male and female mice. However, since alterations, if present, occurred in a gender-independent manner (not shown), it was decided to pool all data in male and female mice.

## 3. Results

### 3.1. General Health Features

There were no visible signs of decline in SMC-KO mice until the age of 20 to 22 weeks. At 23 to 25 weeks, the mice exhibited a mean body weight decrease of 20% compared to WT mice (Supplemental Table S3) and were sacrificed according to the ethical requirements of the EU directive.

### 3.2. Blood Pressure

**Figure 1:**
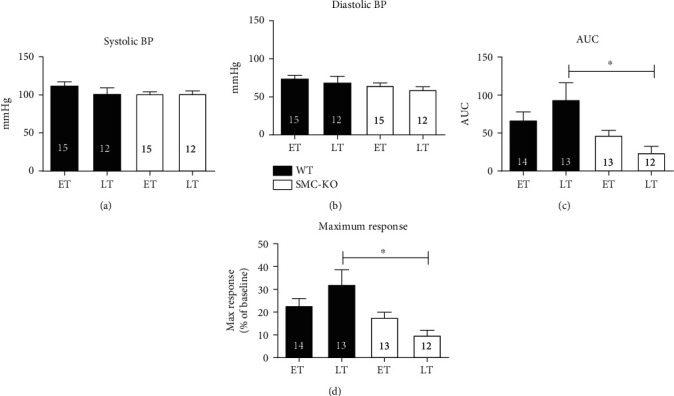
Systolic BP (a) and diastolic BP (b) and functional differences between skin reperfusion after 2 minutes of occlusion in the calculated area under the curve (c) and average maximum response (d) between WT and SMC-KO at ET and LT. The number in each column represents the number of animals in the corresponding group. Statistical differences were analyzed two-way ANOVA followed by Bonferroni's post hoc test (^∗^*p* < 0.05).

Blood pressure measurements showed no significant difference between WT and SMC-KO mice for both ET and LT, neither within the groups of WT and SMC-KO mice nor at different time-points (Figures 1(a) and 1(b)).

### 3.3. Microvascular Vasodilator Function *In Vivo*

At ET, there was no significant difference in reactive hyperemia (indicated by AUC and maximum response) in the hind limb skin between SMC-KO and WT mice (Figures 1(c) and 1(d)). At LT, SMC-KO mice showed significant decreased reactive hyperemia compared to WT mice (Figures 1(c) and 1(d)). When passing from ET to LT, reactive hyperemia tended to increase in WT mice, whereas it tended to decrease in SMC-KO mice.

### 3.4. Mechanical Properties and Dimensions of the Vascular Wall

**Figure 2:**
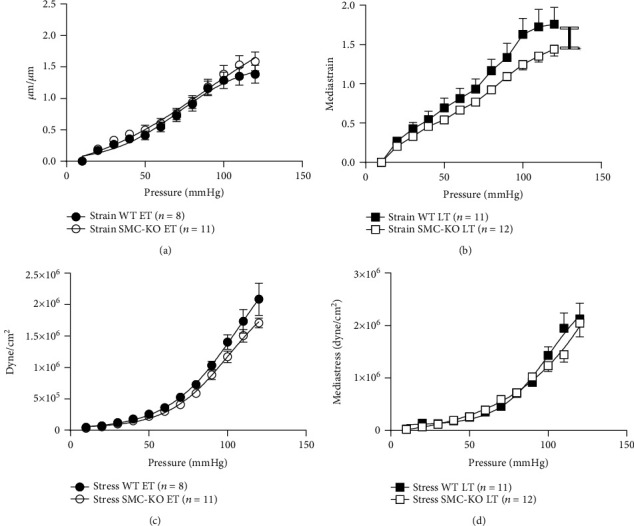
Strain difference at ET (a) and LT (b) and stress differences at ET (c) and LT (d) of the carotid arteries of SMC-KO vs. WT. Statistical difference was analyzed by general linear model repeated measures (^∗^*p* < 0.05).

At LT, SMC-KO mice displayed significantly declined media strain versus WT mice (Figure 2(b)) at comparable media stress (Figures 2(c) and 2(d)), indicating higher stiffness. This was not observed at ET (Figure 2(a)).

### 3.5. Cardiac Function

**Figure 3:**
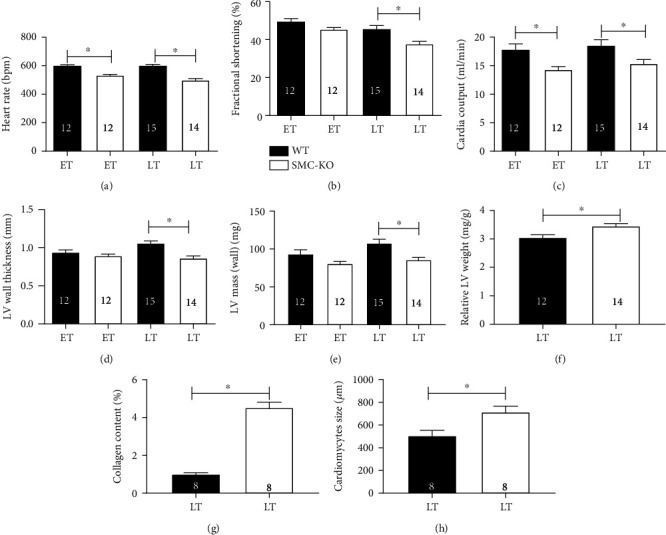
Cardiac function comparison between WT and SMC-KO at ET and LT for heart rate (a), fractional shortening (b), cardiac output (c), LV wall thickness (d), LV mass (e), relative LV weight to body weight at LT (f), LV free wall collagen content (g), and LT free wall cardiomyocyte size (h). The number in each column represents the number of animals in the corresponding group. Statistical differences were analyzed by two-way ANOVA followed by Bonferroni's post hoc test for (a–e) and two-tailed *t*-test for (f–h) (^∗^*p* < 0.05).

Heart rate was lower in SMC-KO mice, both at ET and LT (Figure 3(a)). This was also true for cardiac output (Figure 3(c)). Fractional shortening was significantly lower at LT in SMC-KO mice (Figure 3(b)). At LT, LV wall thickness in SMC-KO mice was significantly smaller compared to WT mice (Figure 3(d)), and the same was true for LV mass (Figure 3(e)). Yet, there were no significant differences for LV mass (Figure 3(e)) and wall thickness at ET (Figure 3(d)) between WT and SMC-KO mice. There were no significant gender differences between males and females in terms of the abovementioned parameters using three-way ANOVA.

Echocardiographic software predicted SMC-KO mice to have a different heart volume and weight as compared to WT. This was confirmed when measuring relative LV weight (=LV weight/body weight) at the age of 25 weeks (Figure 3(f)). Sirius red and Gomori staining (Supplemental Figure S1 A–D) revealed elevated interstitial collagen levels and an increased cardiomyocyte size, respectively, at LT in SMC-KO vs. WT mice (Figures 3(g) and 3(h)). There were no gender differences in both collagen content and cardiomyocyte size using two-way ANOVA.

### 3.6. *Ex Vivo* Vascular Function Assessment

#### 3.6.1. Endothelium-Dependent and Endothelium-Independent Response in SMC-KO Mice versus WT

Iliac U46619 preconstriction values in WT vs. SMC-KO for ET and LT were 3.67 ± 0.25 vs. 3.01 ± 0.31 and 3.28 ± 0.3 vs. 2.32 ± 0.2, respectively. Aortic U46619 preconstriction values in WT vs. SMC-KO for ET and LT were 5.58 ± 1.12 vs. 6.87 ± 0.61 and 7.71 ± 0.85 vs. 7.27 ± 0.86, respectively. Aortic KCl 30 mmol/L preconstriction values in WT vs. SMC-KO for ET and LT were 3.25 ± 0.65 vs. 3.76 ± 0.27 and 3.99 ± 0.45 vs. 3.20 ± 0.22, respectively. All the preconstriction values are in millinewton.

**Figure 4:**
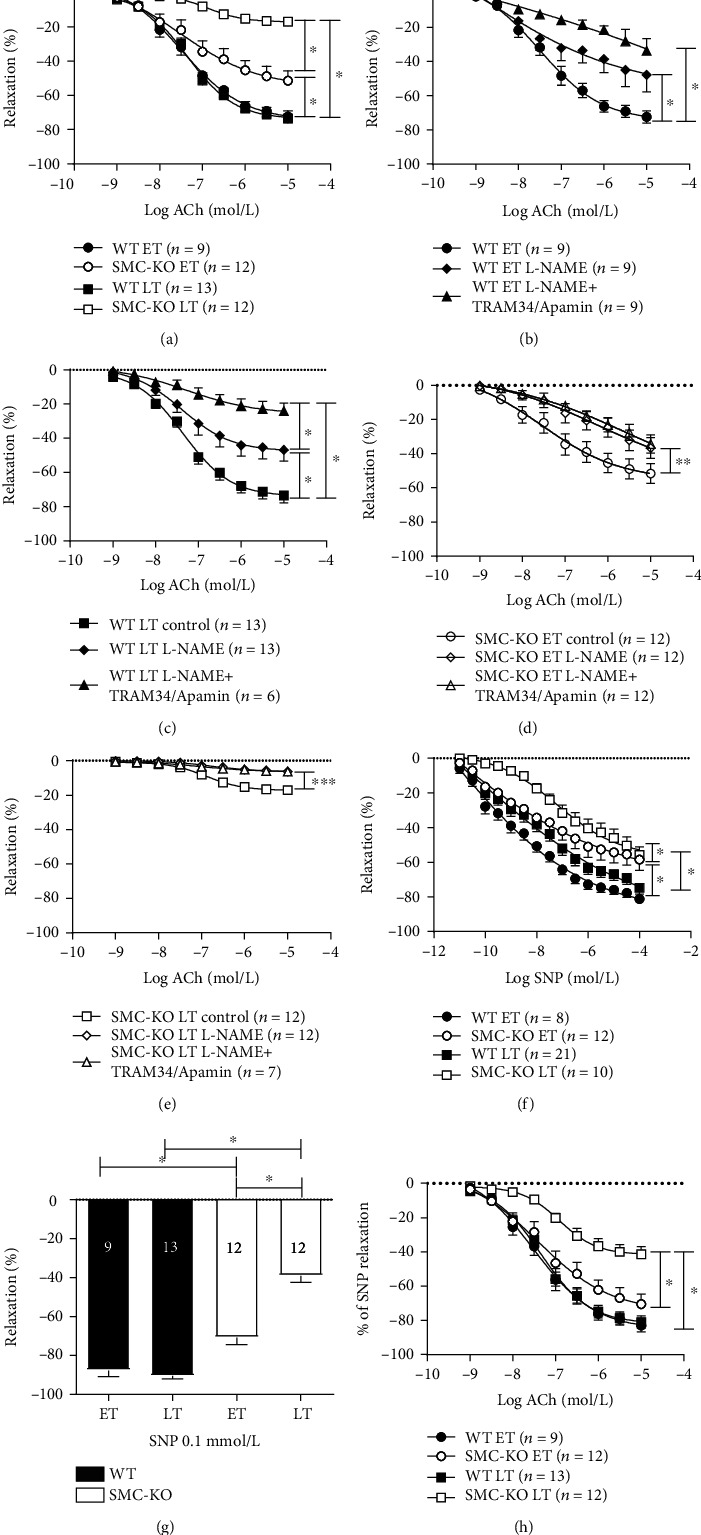
Vasorelaxation in aortic rings of SMC-KO and WT mice for both time-points in response to ACh (10^−9^ to 10^−5^ mol/L) (a). The contribution of NO-cGMP and EDH pathway in WT ET (b), WT LT (c), SMC-KO ET (d), and SMC-KO LT (e). Vasorelaxation in aortic rings of SMC-KO and WT mice for both time-points in response to SNP (10^−11^ to 10^−4^ mol/L) (f), SNP (0.1 mmol/L) after ACh CRC (g), and ACh (10^−9^ to 10^−5^ mol/L) corrected to SNP (0.1 mmol/L) (h). The number in each column represents the number of animals in the corresponding group. Statistical differences were analyzed by general linear model repeated measures for (a–f) and (h) and two-way ANOVA followed by Bonferroni's post hoc test for (g) (^∗^*p* < 0.05).

Cre recombinase expression alone did not alter aortic responses to ACh and SNP (Supplemental Figures S2A and S2B). In contrast, the aortic ACh response showed a decline in SMC-KO mice for both time-points, and at LT, a major part of this response was lost compared to WT littermates (Figure 4(a)). In iliac arteries, a similar decline was observed at LT but not at ET (Supplemental Figure S3A). Inhibition of NOS and EDH in aortic segments revealed that the majority of the response to ACh in WT mice is mediated by NO, while the contribution of EDH was modest (Figures 4(b) and 4(c)). In the SMC-KO mice, EDH was absent at ET already, while the NO-cGMP response showed a less robust decline (Figures 4(d) and 4(e)).

There was a reduction in SNP response for the SMC-KO mice at ET which was further decreased at LT compared with the corresponding WT littermates (Figure 4(f)). When studying the relaxation to a single SNP dose after the ACh CRC, both in aortic (Figure 4(g)) and iliac rings (Figure S3B), the same observation was made.

To explore whether the decline in vasorelaxation is VSMC-dependent, we corrected the ACh results in nonpretreated rings for the response to SNP (0.1 mmol/L) administered after the ACh CRC. In both the aorta and iliac artery, the ACh response in SMC-KO mice was decreased only at LT when correcting for SNP (Figure 4(h) and Figure S3C).

**Figure 5:**
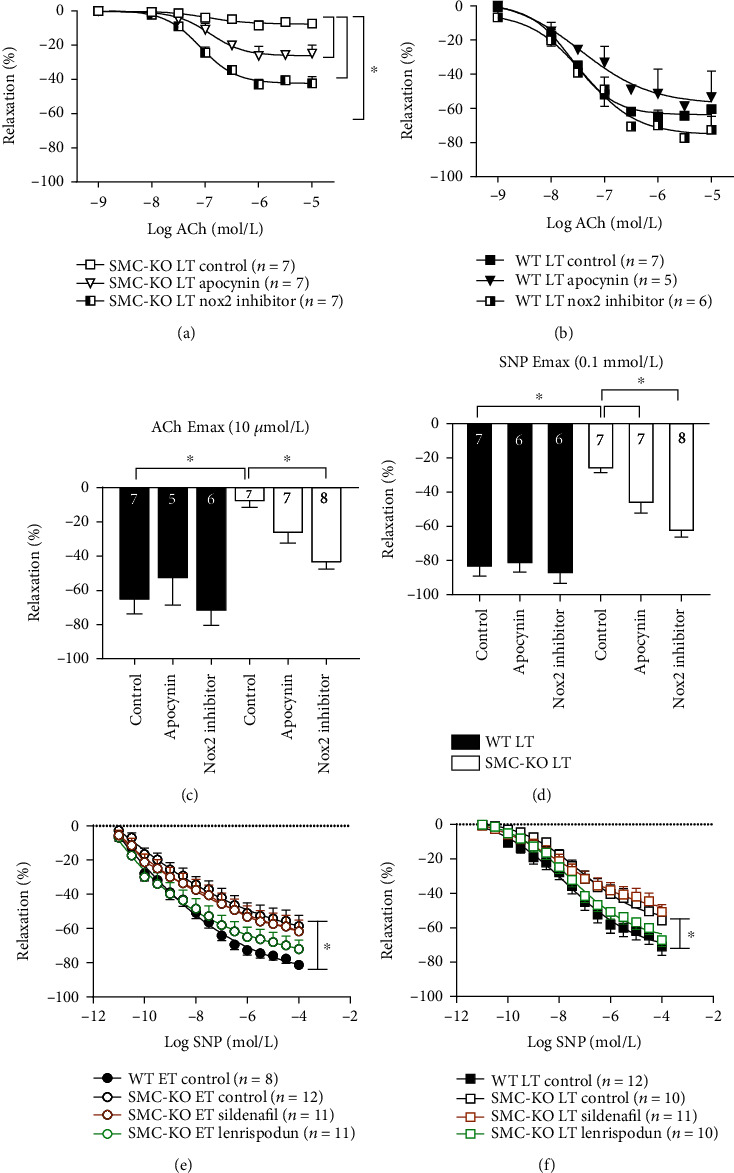
Vasorelaxation response to ACh (10^−9^ to 10^−5^ mol/L) in aortic rings either without inhibitor or with apocynin or GSK279503 preincubation in WT LT (a), SMC-KO LT (b), ACh (10 *μ*mol/L) Emax (c), and SNP (0.1 mmol/L) Emax (d) at LT. Vasorelaxation response to SNP (10^−11^ to 10^−4^ mol/L) in aortic rings either without inhibitor or with sildenafil or lenrispodun preincubation at ET (e) and LT (f). The number in each column represents the number of animals in the corresponding group. Statistical differences were analyzed by general linear model repeated measures for (a), (b), (e), and (f) and one-way ANOVA followed by Bonferroni's post hoc test for (c) and (d) (^∗^*p* < 0.05).

Apocynin and GSK279503 comparably improved ACh and SNP aortic responses in LT SMC-KO mice (Figures 5(a), 5(c), and 5(d)). There was no effect on aortic responses in LT WT mouse rings (Figures 5(b)–5(d)).

After preincubation with lenrispodun in aortic rings, the difference in SNP responses in SMC-KO control vs. lenrispodun at ET and LT was at the borderline to be significant (ET: *p* = 0.1, LT: *p* = 0.08). Moreover, the SNP responses in SMC-KO were identical to those in WT, both in ET and LT mice (Figures 5(e) and 5(f)). No such effects were observed for sildenafil (Figures 5(e) and 5(f)). Neither PDE inhibitor affected SNP responses in WT mice at any time-point (data not shown).

### 3.7. Molecular Analysis

**Figure 6:**
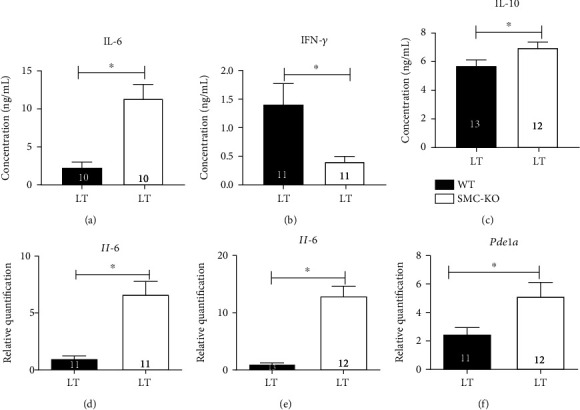
MSD analysis of plasma samples in LT WT and SMC-KO for IL-6 (a), IFN-*γ* (b), and IL-10 (c). qPCR analysis in WT and SMC-KO for Il-6 in LV (d), Il-6 in kidney (e), and Pde1a in the abdominal aorta (f). The number in each column represents the number of animals in the corresponding group. Statistical differences were analyzed by two-tailed *t*-test (^∗^*p* < 0.05).

One of the major changes that occur during ageing is the dysregulation in inflammatory status of cells; thus, we measured certain pro- and anti-inflammatory cytokines in LT SMC-KO mice and the corresponding WT [27]. The plasma protein levels of IL-6 and IL-10 at LT were increased in SMC-KO versus LT mice, and the opposite was true for IFN-*γ* (Figures 6(a)–6(c)). No changes were observed in plasma IL-1*β*, TNF-*α*, IL-2, and IL-4 (data not shown; *n* = 12 for both WT and SMC-KO mice). *Il-6* mRNA expression was upregulated in both the heart (Figure 6(d)) and kidney (Figure 6(e)), and the same was true for *Pde1a* in the aorta (Figure 6(f)). *Pde1a* mRNA expression in the heart and kidney was unaltered (data not shown; *n* = 12 for both WT and SMC-KO mice). *Ercc1* mRNA expression was significantly lower in the abdominal aorta of SMC-KO vs. WT at LT (Figure S4A). DNA damage response and senescent marker *p16* and *p21* expressions were significantly higher in the abdominal aorta of SMC-KO vs. WT at LT (Figures S4B and S4C).

## 4. Discussion

We investigated the role of smooth muscle cell-specific DNA repair deficiency on cardiovascular function in a mouse model with specific loss of *Ercc1* in smooth muscle cells. We found that there was no BP differences between SMC-KO and WT mice nor within SMC-KO mice tested at different time-points. However, LDPI microvascular perfusion imaging showed a progressive decline in reactive hyperemia between ET and LT in SMC-KO mice. Moreover, we evaluated the mechanical properties of carotid arteries and found a significantly diminished compliance between these two groups. Another well-known vascular ageing feature of SMC-KO mice was decreased NO-mediated vasodilation. The latter appeared to be due, at least in part, to upregulation of *Pde1* and *Nox2*. The fact that these vascular alterations did not result in a rise in blood pressure is suggestive of an adaptive response, most likely at the level of the heart. Indeed, at ET, heart rate and cardiac output are lower in the absence of contractile cardiac dysfunction, suggesting an adaptation in autonomic regulation. At LT, however, fractional shortening is reduced, and this was associated with aberrant cardiac remodeling, characterized by an increased collagen content and cardiomyocyte size. Such changes rather point at a malignant cardiac remodeling. This ageing-like phenotype was accompanied by an altered inflammatory status. No gender-specific effects were detected in any of the variables.

As we found a markedly diminished vascular function *in vivo* by LDPI, we suspected impaired vasodilator responses. We demonstrated this in ex vivo organ bath experiments and explored the underlying mechanisms. NO signaling was found to be decreased, evidenced by a reduced response to the NO donor SNP. This reduction was already maximal at ET, and no further decrease was observed thereafter (Figure 4). Importantly, correction of the ACh response to SNP did not normalize the disturbed endothelium-dependent responses in SMC-KO mice to the level of WT (Figure 4; Figure S3). This is indicative of a residual loss of endothelial function. Indeed, no IKCa/SKCa-mediated EDH-dependent response could be observed at both ET and LT in the SMC-KO mice (Figure 4), while the endothelial NO-mediated response additionally diminished over time. Taken together, SMC-KO mice display EDH loss and reduced responsiveness to NO released by the endothelium at ET and LT. Other EDH mechanisms, like BKCa or connexin-mediated effects [28], might also play a role. Therefore, the further unraveling of EDH changes in this accelerated SMC ageing model is warranted.

Decreased NO signaling is a commonly observed feature of mouse models of accelerated ageing caused by reduced functioning of DNA repair proteins [1]. Whole body *Ercc1*^*Δ*/-^ mice are characterized by a combination of a reduced ageing vasodilator response to NO and endothelial dysfunction [29], while in endothelium-specific *Ercc1* KO mice (EC-KO), only the latter was the case [7]. We observed a reduced vasodilator response of VSMC to exogenous NO and to endothelium-derived NO in SMC-KO mice.

The lost NO responsiveness in SMC-KO mice in all likelihood indicates that DNA damage in VSMC leads to decreased bioavailability of NO. The observation that the PDE1 inhibitor, lenrispodun, fully restored the SNP responses in the aorta (Figures 5) suggests that lost NO responsiveness involves *Pde1* upregulation. The aortic *Pde1A* mRNA expression data confirms this view, while a similar *Pde1A* upregulation was observed earlier in whole body *Ercc1*^*Δ*/-^ mice and ageing human VSMC [30]. PDE5 inhibition with sildenafil was without effect in SMC-KO mouse vessels, arguing against a role for *Pde5* upregulation. The reduced endothelial NO release might be explained on the basis of NO inactivation by oxygen radicals, given our finding that Nox inhibition additionally restored the effects of NO. Since selective Nox2 inhibition and nonselective Nox inhibition yielded the same effect, the most likely contributor to the NO-inactivating radicals is Nox2. *Nox2* upregulation is a well-known consequence of cytokine dysregulation [31], evidenced in this model by *Il-6* upregulation that may induce *Nox2* upregulation and ROS production. This process would be expected to eventually lead to reduced NO availability [32]. More general in cardiovascular ageing, Song et al. measured the inflammatory response in cultured aortic vascular smooth muscle cells of aged (16-18 month) vs. young (2-4 months) mice in a nonstimulatory condition and found that aged mice exhibited elevation of basal IL-6. Elevated IL-6 functions in an autocrine manner to further accelerate inflammatory responses in VSMC, thereby making the vasculature prone to atherosclerosis and a less contractile phenotype [33, 34].

Cytokines are known to be altered in *Ercc1*-mutant mice, with specificity for the target cell in which the DNA repair was affected [13, 35, 36]. In SMC-KO mice, circulating IL-10 was found to be increased, while circulating IFN-*γ* was decreased. This might reflect an anti-inflammatory feedback response to the increased plasma IL-6 levels. The IFN-*γ* reduction is in agreement with changes in aged VSMC of *Macaca mulatta* [37], although IL-10 was also reduced in this model. An intricate shift in inflammatory factors has been observed in *Ercc1*-mutant mice and other DNA repair mutants. Therefore, the specific meaning of cytokine shifts is to be interpreted with caution, although a general assumption is that *Ercc1*-mutant mice display primarily a proinflammatory phenotype [38]. In conclusion, the inflammatory status in SMC-KO might be involved in the vascular dysfunction that was observed, and our current observations warrant further inspection in future studies, which would require the development of proper tools for this purpose.

Blood pressure was normal in SMC-KO mice, and at first glance, this is in contradiction with the reduced vasodilation capacity. However, there was a decrease in heart rate and fractional shortening, and cardiac output was decreased by 20% on average in SMC-KO vs. WT mice. At an equal level of vascular resistance in the two mouse strains, this should translate to a 20% lower blood pressure in the SMC-KO mice (blood pressure = cardiac output × vascular resistance). Yet, the blood pressure was identical in both strains. Reasoning backward to how this translates to vascular resistance, the Hagen-Poiseuille equation can be applied. It states that pressure or flow is changed with blood vessel length, blood viscosity, and radius^4^. Vessel length and blood viscosity being similar and with blood flow (=cardiac output) being reduced by 20%, the vascular diameter of SMC-KO should on average be 0.95x that of WT mice. Since this requires a state of vasoconstriction relative to WT, SMC-KO might be able to sustain a normal blood pressure despite the strongly reduced vasodilation capacity, e.g., by reducing sympathetic neurohormonal input. The reduced cardiac output, if due to such regulation (or due to pathological remodeling or a combination of both), therefore accommodates normalization of blood pressure in SMC-KO mice. Like in the vasculature, the IL-6 increase might be involved in cardiac changes. Interestingly, Meléndez et al. showed in rats that IL-6 infusion resulted in concentric LV remodeling, a significant increase in collagen volume fraction and relative increases in cardiomyocyte width and length that all were independent of blood pressure changes [39]. Despite the remodeling, no overt heart failure was observed in SMC-KO mice. It seems likely that apart from pathological remodeling, adaptation of hemodynamic function by neurohormonal mechanisms accounts for the observed hemodynamic changes. However, the possibility of heart failure like phenotype cannot be excluded and needs further investigation.

In conclusion, SMC-KO mice show a progressive ageing phenotype in resistant and conduit arteries. The changes induced by DNA damage might be limited to VSMC, although it seems that dysfunction in VSMC eventually has an impact on EC function as well, affecting the bioavailability of endothelium-derived NO through Nox2-mediated ROS production. PDE1 inhibition restores vasodilator function, whereas PDE5 seems to play a minor role in progressed VSMC dysfunction. As a future perspective, it might be of interest to study the effect of chronic treatment with PDE1 specific inhibitors on progression of vascular dysfunction, inflammation, and cardiac remodeling. Currently, lenrispodun is under clinical development for treatment of neurodegenerative disease and has been shown to be a safe and well-tolerated drug in heart failure patients [40].

## Data Availability

Data is available on request.

## Supplementary Materials

Supplementary Materials Supplementary 1. Table S1: TaqMan ID assay and probe context sequence that were used for *Pde1a*, *Il-6*, and *Gapdh* qPCR measurements. Table S2: sense and antisense sequence of primers that were used for *p16*, *p21*, *Ercc1*, *β-actin*, and *Hprt1* qPCR measurements. Supplementary 2. Table S3: body weight of SMC-KO and WT mice stratified by age and gender. Supplementary 3. Figure S1: photographs of collagen staining for WT (A) and SMC-KO (B) and cardiomyocyte staining for WT (C) and SMC-KO (D) at LT. Supplementary 4. Figure S2: vasorelaxation response to ACh (10^−9^ to 10^−5^ mol/L) (A) and SNP (10^−11^ to 10^−4^ mol/L) (B) in aortic rings of WT mice with and without Cre. Statistical differences were analyzed by general linear model repeated measures. Supplementary 5. Figure S3: vasorelaxation response to ACh (10^−9^ to 10^−5^ mol/L) (A), SNP (0.1 mmol/L) after ACh (B), and ACh (10^−9^ to 10^−5^ mol/L) corrected for SNP (0.1 mmol/L) in iliac rings of SMC-KO and WT mice for both time-points. The number in each column represents the number of animals in the corresponding group. Statistical differences were analyzed by general linear model repeated measures for A and C and two-way ANOVA followed by Bonferroni's post hoc test for B (^∗^*p* < 0.05). Supplementary 6. Figure S4: qPCR analysis in WT and SMC-KO in abdominal aorta at LT for *Ercc1* (A), *p16* (B), and *p21* (C). The number in each column represents the number of animals in the corresponding group. Statistical differences were analyzed by two-tailed *t*-test (^∗^*p* < 0.05).

## References

[1] Golshiri K., Ataei Ataabadi E., Portilla Fernandez E. C., Jan Danser A., Roks A. J. (2020). The importance of the nitric oxide-cGMP pathway in age-related cardiovascular disease: focus on phosphodiesterase-1 and soluble guanylate cyclase. *Basic & Clinical Pharmacology & Toxicology*.

[2] Lakatta E. G., Levy D. (2003). Arterial and cardiac aging: major shareholders in cardiovascular disease enterprises. *Circulation*.

[3] López-Otín C., Blasco M. A., Partridge L., Serrano M., Kroemer G. (2013). The hallmarks of aging. *Cell*.

[4] del Campo L., Sánchez-López A., González-Gómez C., Andrés-Manzano M. J., Dorado B., Andrés V. (2020). Vascular smooth muscle cell-specific progerin expression provokes contractile impairment in a mouse model of Hutchinson-Gilford progeria syndrome that is ameliorated by nitrite treatment. *Cell*.

[5] Kovacic J. C., Moreno P., Nabel E. G., Hachinski V., Fuster V. (2011). Cellular senescence, vascular disease, and aging: part 2 of a 2-part review: clinical vascular disease in the elderly. *Circulation*.

[6] Garinis G. A., GTJ H., Vijg J., JHJ H. (2008). DNA damage and ageing: new-age ideas for an age-old problem. *Nature Cell Biology*.

[7] Bautista-Niño P. K., Portilla-Fernandez E., Rubio-Beltrán E. (2020). Local endothelial DNA repair deficiency causes aging-resembling endothelial-specific dysfunction. *Clinical Science*.

[8] Yousefzadeh M. J., Zhao J., Bukata C. (2020). Tissue specificity of senescent cell accumulation during physiologic and accelerated aging of mice. *Aging Cell*.

[9] Vermeij W. P., Hoeijmakers J. H. J., Pothof J. (2016). Genome integrity in aging: human syndromes, mouse models, and therapeutic options. *Annual Review of Pharmacology and Toxicology*.

[10] Houtsmuller A. B., Rademakers S., Nigg A. L., Hoogstraten D., Hoeijmakers J. H., Vermeulen W. (1999). Action of DNA repair endonuclease ERCC1/XPF in living cells. *Science*.

[11] Ahmad A., Robinson A. R., Duensing A. (2008). ERCC1-XPF endonuclease facilitates DNA double-strand break repair. *Molecular and Cellular Biology*.

[12] Bergstralh D. T., Sekelsky J. (2008). Interstrand crosslink repair: can XPF-ERCC1 be let off the hook?. *Trends in Genetics*.

[13] Niedernhofer L. J., Garinis G. A., Raams A. (2006). A new progeroid syndrome reveals that genotoxic stress suppresses the somatotroph axis. *Nature*.

[14] Vermeij W. P., Dollé M. E., Reiling E. (2016). Restricted diet delays accelerated ageing and genomic stress in DNA-repair- deficient mice. *Nature*.

[15] Wu H., van Thiel B. S., Bautista-Niño P. K. (2017). Dietary restriction but not angiotensin II type 1 receptor blockade improves DNA damage-related vasodilator dysfunction in rapidly aging Ercc1*Δ*/− mice. *Clinical Science*.

[16] Golshiri K., Ataei Ataabadi E., Brandt R. (2020). Chronic sildenafil treatment improves vasomotor function in a mouse model of accelerated aging. *International Journal of Molecular Sciences*.

[17] Durik M., Kavousi M., van der Pluijm I. (2012). Nucleotide excision DNA repair is associated with age-related vascular dysfunction. *Circulation*.

[18] Dollé M. E., Kuiper R. V., Roodbergen M. (2011). Broad segmental progeroid changes in short-livedErcc1−/*Δ*7mice. *Pathobiology of Aging & Age-related Diseases*.

[19] Zhang J., Zhong W., Cui T. (2006). Generation of an adult smooth muscle cell-targeted Cre recombinase mouse model. *Arteriosclerosis, Thrombosis, and Vascular Biology*.

[20] Chakraborty R., Saddouk F. Z., Carrao A. C., Krause D. S., Greif D. M., Martin K. A. (2019). Promoters to study vascular smooth muscle. *Arteriosclerosis, Thrombosis, and Vascular Biology*.

[21] Doig J., Anderson C., Lawrence N., Selfridge J., Brownstein D., Melton D. (2006). Mice with skin-specific DNA repair gene (Ercc1) inactivation are hypersensitive to ultraviolet irradiation-induced skin cancer and show more rapid actinic progression. *Oncogene*.

[22] el-Bizri N., Guignabert C., Wang L. (2008). SM22*α*-targeted deletion of bone morphogenetic protein receptor 1A in mice impairs cardiac and vascular development, and influences organogenesis. *Development*.

[23] Resch M., Wiest R., Moleda L. (2009). Alterations in mechanical properties of mesenteric resistance arteries in experimental portal hypertension. *American Journal of Physiology-Gastrointestinal and Liver Physiology*.

[24] van Deel E. D., de Boer M., Kuster D. W. (2011). Exercise training does not improve cardiac function in compensated or decompensated left ventricular hypertrophy induced by aortic stenosis. *Journal of Molecular and Cellular Cardiology*.

[25] Au-Bridges L. E., Au-Williams C. L., Au-Pointer M. A., Au-Awumey E. M. (2011). Mesenteric artery contraction and relaxation studies using automated wire myography. *Journal of Visualized Experiments*.

[26] Li P., Zheng H., Zhao J. (2016). Discovery of potent and selective inhibitors of phosphodiesterase 1 for the treatment of cognitive impairment associated with neurodegenerative and neuropsychiatric diseases. *Journal of Medicinal Chemistry*.

[27] Chung H. Y., Kim D. H., Lee E. K. (2019). Redefining chronic inflammation in aging and age-related diseases: proposal of the senoinflammation concept. *Aging and Disease*.

[28] Goto K., Ohtsubo T., Kitazono T. (2018). Endothelium-dependent hyperpolarization (EDH) in hypertension: the role of endothelial ion channels. *International Journal of Molecular Sciences*.

[29] Bautista-Niño P. K., Portilla-Fernandez E., Vaughan D. E., Danser A., Roks A. J. (2016). DNA damage: a main determinant of vascular aging. *International Journal of Molecular Sciences*.

[30] Niño P. K. B., Durik M., Danser A. H. (2015). Phosphodiesterase 1 regulation is a key mechanism in vascular aging. *Clinical Science*.

[31] Sena C. M., Leandro A., Azul L., Seiça R., Perry G. (2018). Vascular Oxidative Stress: Impact and Therapeutic Approaches. *Frontiers in Physiology*.

[32] Didion S. P. (2017). Cellular and oxidative mechanisms associated with interleukin-6 signaling in the vasculature. *International Journal of Molecular Sciences*.

[33] Yang S., Hua S., Dominik S., Peiying S., Patty J. L., Daniel R. G. (2012). Aging enhances the basal production of IL-6 and CCL2 in vascular smooth muscle cells. *Arteriosclerosis, Thrombosis, and Vascular Biology*.

[34] Park M., Choi S., Kim S. (2019). NF-*κ*B-responsive miR-155 induces functional impairment of vascular smooth muscle cells by downregulating soluble guanylyl cyclase. *Experimental & Molecular Medicine*.

[35] de Waard M. C., van der Pluijm I., Zuiderveen Borgesius N. (2010). Age-related motor neuron degeneration in DNA repair-deficient Ercc1 mice. *Acta Neuropathologica*.

[36] Karakasilioti I., Kamileri I., Chatzinikolaou G. (2013). DNA damage triggers a chronic autoinflammatory response, leading to fat depletion in NER progeria. *Cell Metabolism*.

[37] Csiszar A., Sosnowska D., Wang M., Lakatta E. G., Sonntag W. E., Ungvari Z. (2012). Age-associated proinflammatory secretory phenotype in vascular smooth muscle cells from the non-human primate Macaca mulatta: reversal by resveratrol treatment. *The Journals of Gerontology: Series A*.

[38] Kim D. E., Dollé M. E. T., Vermeij W. P. (2020). Deficiency in the DNA repair protein ERCC1 triggers a link between senescence and apoptosis in human fibroblasts and mouse skin. *Aging Cell*.

[39] Meléndez G. C., McLarty J. L., Levick S. P., du Y., Janicki J. S., Brower G. L. (2010). Interleukin 6 mediates myocardial fibrosis, concentric hypertrophy, and diastolic dysfunction in rats. *Hypertension*.

[40] Gilotra N. A., Devore A., Hays A. (2020). Cardiac and hemodynamic effects of acute phosphodiesterase-1 inhibition in human heart failure. *Journal of Cardiac Failure*.

