# Supplementary material for: Vascular Ageing Features Caused by Selective DNA Damage in Smooth Muscle Cell
Source: Oxid Med Cell Longev. 2021 Aug 31;2021:2308317. doi: 10.1155/2021/2308317 (PMC8423575; doi:10.1155/2021/2308317)
Supplement: Supplementary Materials — Supplementary 1. Table S1: TaqMan ID assay and probe context sequence that were used for Pde1a, Il-6, and Gapdh qPCR measurements. Table S2: sense and antisense sequence of primers that were used for p16, p21, Ercc1, β-actin, and Hprt1 qPCR measurements. Supplementary 2. Table S3: body weight of SMC-KO and WT mice stratified by age and gender. Supplementary 3. Figure S1: photographs of collagen staining for WT (A) and SMC-KO (B) and cardiomyocyte staining for WT (C) and SMC-KO (D) at LT. Supplementary 4. Figure S2: vasorelaxation response to ACh (10−9 to 10−5 mol/L) (A) and SNP (10−11 to 10−4 mol/L) (B) in aortic rings of WT mice with and without Cre. Statistical differences were analyzed by general linear model repeated measures. Supplementary 5. Figure S3: vasorelaxation response to ACh (10−9 to 10−5 mol/L) (A), SNP (0.1 mmol/L) after ACh (B), and ACh (10−9 to 10−5 mol/L) corrected for SNP (0.1 mmol/L) in iliac rings of SMC-KO and WT mice for both time-points. The number in each column represents the number of animals in the corresponding group. Statistical differences were analyzed by general linear model repeated measures for A and C and two-way ANOVA followed by Bonferroni's post hoc test for B (∗p < 0.05). Supplementary 6. Figure S4: qPCR analysis in WT and SMC-KO in abdominal aorta at LT for Ercc1 (A), p16 (B), and p21 (C). The number in each column represents the number of animals in the corresponding group. Statistical differences were analyzed by two-tailed t-test (∗p < 0.05). [file 2308317.f1.docx]

**Supplementary Materials**

**Legends**

**Tables:**

Table S1. Taqman ID assay and probe context sequence that were used for *Pde1a,* *Il-6* and *Gapdh* qPCR measurements.

Table S1. Sense and antisense sequence of primers that were used for *p16*, *p21*, *Ercc1, β-actin* and *Hprt1* qPCR measurements.

Table S3. Body weight of SMC-KO and WT mice stratified by age and gender.

**Figures:**

Figure S1. Photographs of collagen staining for WT (A) and SMC-KO (B) and cardiomyocytes staining for WT (C) and SMC-KO (D) at LT.

Figure S2. Vasorelaxation response to ACh (10^-9^ to 10^-5^ mol/L) (A) and SNP (10^-11^ to 10^-4^ mol/L) (B) in aortic rings of WT mice with and without Cre. Statistical differences were analysed by General linear model repeated measures.

Figure S3. Vasorelaxation response to ACh (10^-9^ to 10^-5^ mol/L) (A), SNP (0.1mmol/L) after ACh (B) and ACh (10^-9^ to 10^-5^ mol/L) corrected for SNP (0.1mmol/L) in iliac rings of SMC-KO and WT mice for both time-points. The number in each column represents the number of animals in the corresponding group. Statistical differences were analysed by General linear model repeated measures for A and C and two-way ANOVA followed by Bonferroni's post hoc test for B (* = *p <* 0.05).

Figure S4. qPCR analysis in WT and SMC-KO in abdominal aorta at LT for *Ercc1* (A), *p16* (B) and *p21* (C). The number in each column represents the number of animals in the corresponding group. Statistical differences were analysed by two-tailed t-test (* = p < 0.05).

**Table S1**

| *Gene* | *Probe context sequence* | *Template ID* | *Amplicon size* |
| --- | --- | --- | --- |
| ***Pde1a*** | ATACAGGTATCATGCACTGGCTCAC | Mm00450244_m1 | *90* |
| ***Il-6*** | TGAGAAAAGAGTTGTGCAATGGCAA | Mm00446190_m1 | *78* |
| ***Gapdh*** | GGTGTGAACGGATTTGGCCGTATTG | Mm99999915_g1 | *107* |

| ***Gene*** | ***Sense*** | ***Antisense*** | ***Template ID*** | | ***Amplicon size*** |
| --- | --- | --- | --- | --- | --- |
| ***Ercc1*** | 5’-CATTTGGATCCCTGGAACAGC-3’ | 5’-TAGTACTTCAAAGAGCCTGCGG-3 | | NM_007948.2 | *102* |
| ***p16*** | 5’-CGCTCTGGCTTTCGTGAACA-3′ | 5’-GTTGCCCATCATCATCACCTGG-3′ | | NM_009877.2 | *95* |
| ***p21*** | 5’-GTCAGGCTGGTCTGCCTCCG-3′ | 5’-CGGTCCCGTGGACAGTGAGCAG-3′ | | NM_007669.5 | *103* |
| ***β-actin*** | 5’-TTCTTGGGTATGGAATCCTGTGG-3′ | 5’-GTCTTTACGGATGTCAACGTCAC-3′ | | NM_007393.5 | *81* |
| ***Hprt1*** | 5’-CCTAAGATGAGCGCAAGTTGAA-3′ | 5’-CCACAGGCTAGAACACCTGCTAA-3′ | | NM_013556.2 | *92* |

**Table S2**

**Table S3**

|  | WT ET male | WT LT male | WT ET female | WT LT female | SMC-KO ET male | SMC-KO LT male | SMC-KO ET female | SMC-KO LT female |
| --- | --- | --- | --- | --- | --- | --- | --- | --- |
| Number of mice | **6** | **6** | **7** | **7** | **7** | **6** | **8** | **8** |
| Mean ± SEM | **29.93± 0.8082** | **33.32 ± 0.6096** | **22.14 ± 0.7792** | **25.06 ± 0.9071** | **27.60 ± 0.4397** | **22.77 ± 0.4667** | **21.51 ± 0.6122** | **20.27 ± 0.3771** |

**Figure S1**

**B**

**A**


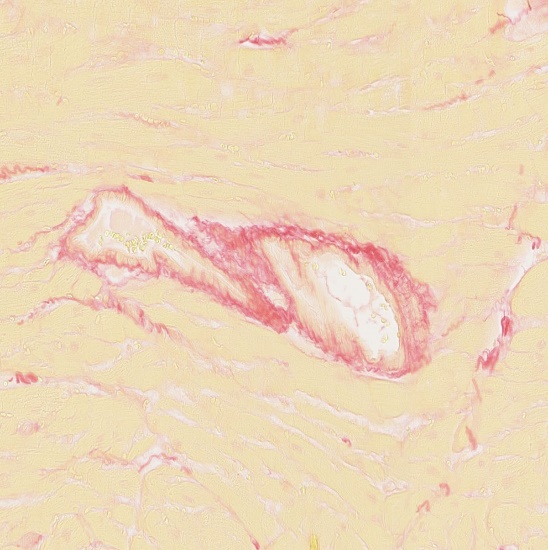

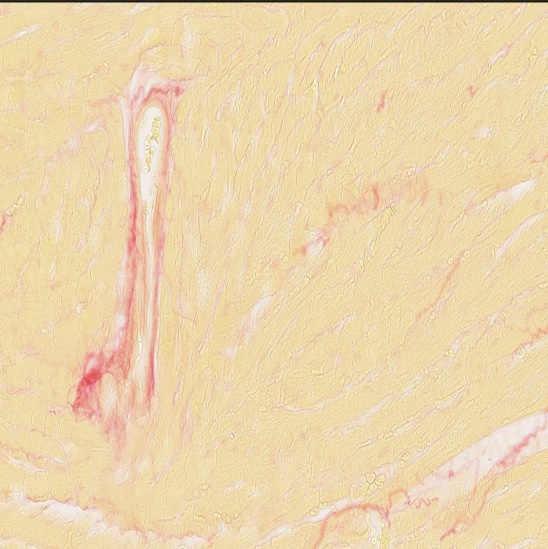


**C**

**D**


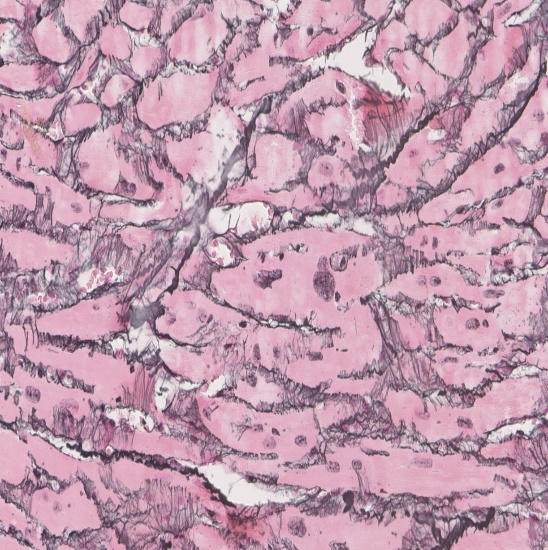

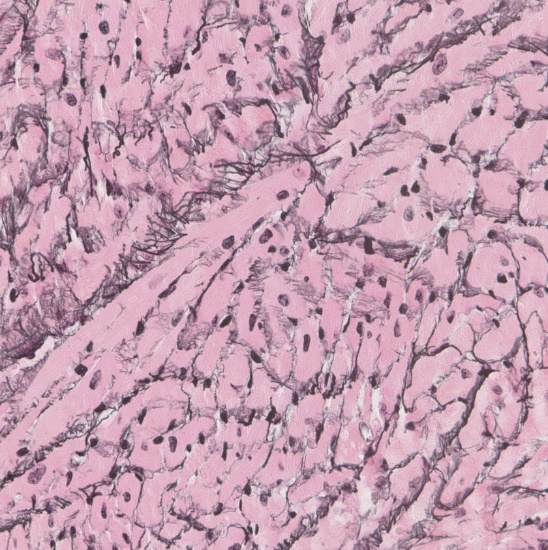


**Figure S3**

**Figure S2**

**B**

**A**

**A**

**Figure S3**

**B**

**A**

**WT**

**SMC-KO**

**C**

**Figure S4**

**SMC-KO LT**

**WT LT**

**C**

**B**

**A**
